# Rapid and Convenient Quantitative Analysis of SARS-CoV-2 RNA in Serous Saliva with a Direct PCR Method

**DOI:** 10.3390/epidemiologia2030023

**Published:** 2021-07-28

**Authors:** Chikaya Deura, Kenji Nakayama

**Affiliations:** Clinical Examination Group, Center for Precision Medicine Supports, Shimadzu Techno-Research, Inc., 7 Nishinokyo-Shimoaicho, Nakagyo-ku, Kyoto 604-8436, Japan; chik-deu@editforce.jp

**Keywords:** SARS-CoV-2, COVID-19, PCR, quantitative analysis, serous saliva, Rayleigh scattering influence

## Abstract

Sensitive and accurate detection of severe acute respiratory syndrome coronavirus 2 (SARS-CoV-2), frequently performed using direct polymerase chain reaction (PCR), is essential for restricting the spread of coronavirus disease 2019 (COVID-19). However, studies evaluating accurate detection are still required. This study evaluated the quantitativeness and sensitivity of the Ampdirect™ 2019-nCoV detection kit, a direct PCR method. Using saliva with or without Tris-buffered saline (TBS) dilution, linearity, and limits of the N1 and N2 regions of SARS-CoV-2 genomic RNA were assessed using EDX SARS-CoV-2 RNA standard dissolved in RNase-free water (RFW). Fluorescence intensities in non-diluted saliva were higher than those in TBS-diluted samples. Linear regression analysis of detected quantification cycle values and spiked standard RNA concentrations showed that the coefficient of determination of the N1 and N2 genes was 0.972 and 0.615 in RFW and 0.947 and 0.660 in saliva, respectively. N1- and N2-positive detection rates in saliva were 46% (6/13 tests) and 0% (0/12 tests) at one copy/reaction, respectively. These results indicate good quantitativeness and sensitivity for N1 but not for N2. Therefore, our findings reveal that the Ampdirect™ 2019-nCoV system, especially targeting the N1 gene, enables rapid and convenient quantification of SARS-CoV-2 RNA in saliva at one copy/reaction.

## 1. Introduction

On 21 July 2021, more than 190 million coronavirus disease 2019 (COVID-19) infections and 4.1 million deaths were reported, and this epidemic had spread to over 207 countries and territories around the world (Weekly Operational Update on COVID-19 - 21 July 2021, reported by the World Health Organization). The detection and isolation of asymptomatic individuals and patients with mild disease are essential for controlling the spread of severe acute respiratory syndrome coronavirus 2 (SARS-CoV-2), because they have a high potential for virus transmission despite low-level viral loads [1,2]. Therefore, high sensitivity and high-accuracy analysis of SARS-CoV-2 is important to restrict the spread of COVID-19 infection. Detection of the SARS-CoV-2 RNA is performed using several protocols such as the loop-mediated isothermal amplification approach (LAMP) [3] and quantitative reverse transcription-polymerase chain reaction (RT-qPCR) with and without nucleotide extraction [4,5,6] in clinical testing of COVID-19. On the other hand, analytical variations have warranted close management by clinical laboratories, especially for quality control in polymerase chain reaction (PCR) testing. Therefore, methods allowing better understanding and interpretation of PCR-test results have become important. PCR testing can be interpreted using quantitative, qualitative, and semi-quantitative evaluation methods. Therefore, daily clinical testing requires evaluation with sufficient understanding of test quality [7,8,9].

Saliva is an attractive sample source for detecting SARS-CoV-2 infections. It is easy to self-collect, and therefore reduces the exposure risk for the collector, unlike nasopharyngeal swab (NSP) collection. Moreover, a higher viral load is found in saliva samples than in NSP samples from COVID-19 patients [10,11,12,13]. In addition, when confirming the website of the GTEx portal [14] according to a report by Xu et al. [15], the expression of angiotensin-converting enzyme 2 (ACE2) in human salivary glands (i.e., parotid, submandibular, and sublingual glands) is higher than that in the lungs. Serous saliva is assumed to become an effective clinical specimen for detecting SARS-CoV-2 because it is mainly secreted from the parotid gland. Although a non-purification real-time PCR method (i.e., a direct PCR method) is useful for rapid and simple testing of SARS-CoV-2 [16,17], few studies have examined its sensitivity or quantitativeness. This study aims at establishing a highly sensitive and quantitative detection of SARS-CoV-2 from saliva samples using a direct PCR method.

## 2. Materials and Methods

### 2.1. Ethics Statement

This study was conducted with the approval (R-2020-001) of the Ethics Committee of Shimadzu Techno-Research, Inc. (Nakagyou-ku, Kyoto, Japan). Clinical materials were used after written informed consent was obtained and approved according to protocols by the Institutional Review Board of Shimadzu Techno-Research.

### 2.2. Collection and Pretreatment of Saliva

Human saliva samples were collected from eight healthy volunteers (two women and six men; age-ranges: 38–65 years old) from Shimadzu Techno-Research, Inc. and placed in 50 mL centrifugation tubes. Samples were centrifuged at 2200× *g* for 5 min at 4 °C, and the supernatants were transferred to 1.5 mL tubes. Two pretreatment protocols for human saliva were tested to investigate a suitable quantitative SARS-CoV-2 detection method with real-time PCR. The first method, called “dilution method” in this report, was as follows: saliva samples were diluted with saline buffered with 10 mM Tris-HCl, pH 8.0 (dilution, 1:3; Kanto Chemical CO., Inc., Tokyo, Japan). After vortexing, the diluted samples were briefly centrifuged at 3000× *g* for 1 min at 4 °C, and the supernatants were used as specimens for real-time PCR analysis. The second method, called the “non-dilution method”, was combined with high-speed centrifugation. After centrifuging at 2200× *g* for 5 min at 4 °C, the saliva samples that had been collected in 1.5 mL tubes were centrifuged at 20,000× *g* for 10 min at 4 °C, and the supernatants (i.e., serous salivary fluids) were used as specimens for real-time PCR analysis.

The dilution method has been widely utilized in Japan. However, this method leads to dilution of the RNA concentration in the original specimens. Therefore, in this study, the dilution method was not combined with high-speed centrifugation or validated to avoid diluting the original saliva.

### 2.3. Real-Time PCR Analysis

In this study, the Ampdirect™ 2019-nCoV detection kit (Shimadzu, Kyoto, Japan) was purchased and used for quantitative real-time PCR analysis of SARS-CoV-2. This kit does not require RNA extraction or purification steps and consists of sample treatment reagent (Str) and Reagents A, B, and C. To extract the SARS-CoV-2 genomic RNA from the viral particles, a 5 μL sample aliquot was thoroughly mixed with the same volume (5 μL) of Str solution containing sodium hydroxide as the main component in a PCR tube, and subsequently heated at 90 °C for 5 min. The tubes were centrifuged at 10,000× *g* for 30 s at 4 °C. The three-reagent mixture (Reagents A, B, and C, 15 μL) was added to the heat-treated samples. Reagent A was a buffer solution containing magnesium chloride. Reagent B included a mixture of primers and probes for detecting both the internal positive control (IPC) and the N1 and N2 gene regions of the SARS-CoV-2 genomic RNA. The primer and probe sets for SARS-CoV-2 were designed based on the database accessible from the Centers for Disease Control and Prevention [18] (more detailed sequence information about the primers and probe was not obtained from the Shimadzu Corporation, even after several requests). The fluorescent reagents Cy5 (excitation: 650 nm, emission: 670 nm), FAM (excitation: 495 nm, emission: 520 nm), and ROX (excitation: 575 nm, emission: 600 nm) were also utilized for detecting the IPC, N1, and N2, respectively. A mixture of reverse transcriptase (RT) and DNA polymerase in Reagent C was used for amplification. The direct RT-PCRs were carried out in a solution mixture containing a chemical cocktail for suppressing the effects of both RNases and PCR inhibitors by several biomolecules in the saliva. The IPC comprises synthetic DNA plasmids. Real-time PCR analysis was run on a LightCycler^®^ 96 (Roche, Basel, Switzerland) instrument under the conditions described in Table 1. All data analyses were performed using LightCycler^®^ 96 software (Version 1.1.0.1320, Roche Diagnostics GmbH, Mannheim, Germany). Quantification cycle (Cq) values were obtained and utilized after analyzing the original raw data using the software.

### 2.4. Linear Regression Analyses of the N1 and N2 Analysis in RNase-Free Water and Non-Diluted (Serous) Saliva

To assess the quantitative ability of the Ampdirect™ 2019-nCoV detection kit, the linearity of the SARS-CoV-2 N1 and N2 gene analyses was confirmed using the EDX SARS-CoV-2 standard (Exact Diagnostics, TX, USA). The SARS-CoV-2 standards (STDs) were synthetic RNA transcripts containing SARS-CoV-2 genes, namely, *E*, *N*, *Orf1ab*, *RdRp*, and *S* [19]. Two analyses were designed for confirming the linearity of the SARS-CoV-2 RNA STDs as follows. The first was to analyze the RNA STDs in deionized, distilled DNase- and RNase-free water (RFW; MP Biomedicals, Solon, OH, U.S.A.) with the following concentration gradients: 1000, 800, 600, 400, 200, 100, 50, and 10 copy (cp)/reaction (*n* = 3 per concentration level). The other was to analyze the RNA STDs in the serous saliva with the following concentration gradients: 200, 100, 50, and 10 cp/reaction (*n* = 3 per concentration level). Four microliters of saliva and 5 μL Str solution were mixed in a PCR tube. After heating at 90 °C for 5 min and centrifuging at 10,000× *g* for 30 s at 4 °C, 1 μL of the diluted SARS-CoV-2 RNA STDs was added to the mixture. Thereafter, 15 μL of the three-reagent mixture (Reagents A, B, and C) was added to the PCR tube and mixed thoroughly. A 15 μL volume of the three-reagent mixture was added to the samples, and the RT real-time PCR analyses were subsequently performed using a LightCycler^®^ 96 instrument. To confirm the test results of the N1 and N2 analyses, calibration curves were constructed from the Cq values using Excel software (Microsoft Office 365 ProPlus version 1808).

### 2.5. Detection of SARS-CoV-2 RNA at Low Concentrations

To investigate the detection limit of the N1 and N2 genes using the Ampdirect™ 2019-nCoV detection kit, the amplification of SARS-CoV-2 RNA in RFW and serous saliva samples containing low RNA concentration STDs (10, 5, and 1 cp/reaction, *n* = 12 or 13 per concentration level) was analyzed. Four microliters of saliva and 5 μL Str solution were mixed in a PCR tube. After heating at 90 °C for 5 min and centrifuging at 10,000× *g* for 30 s at 4 °C, 1 μL of the diluted SARS-CoV-2 RNA was added to the mixture. Thereafter, 15 μL of the three-reagent mixture was added to the PCR tube and mixed well. N1 and N2 detections were evaluated using LightCycler^®^ 96 software.

### 2.6. Statistical Analysis

The data are presented as mean ± standard error of measurement. Statistical analyses were performed using R software (version 4.0.2). The Mann–Whitney–Wilcoxon test was used to identify a non-parametric significant difference between two groups. Significance was set at *p* < 0.05.

## 3. Results

### 3.1. Amplification of IPC in Diluted and Non-Diluted Saliva Samples

**Figure 1:**
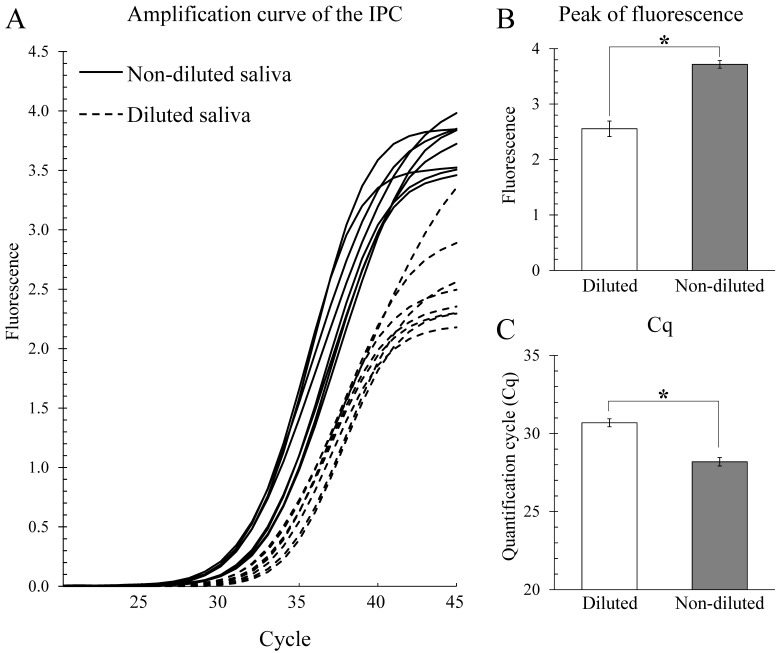
Amplification of the internal positive control (IPC) in diluted and non-diluted saliva. Human saliva samples were collected from eight healthy volunteers. (**A**) Amplification curves of IPC in diluted (dashed line) and non-diluted (solid line) saliva samples. (**B**) Fluorescence intensities of the IPC at the end of each reaction in diluted (open column) and non-diluted (grey column) saliva samples. (**C**) Quantification cycle (Cq) values of the IPC in diluted (open column) and non-diluted (grey column) saliva samples. Data are shown as mean ± SEM (*n* = 8). The Mann–Whitney–Wilcoxon test was used to identify a non-parametric significant difference between diluted and non-diluted saliva samples. Asterisks (*) indicate significant differences at *p* < 0.01 between diluted and non-diluted samples.

The amplification curves of the IPC in diluted and non-diluted saliva are shown in Figure 1A. A clear difference between the IPC amplification curve shapes of the diluted and non-diluted saliva samples was observed. The fluorescence intensities at the end of each reaction in the non-diluted saliva samples (clear serous salivary fluids) were significantly higher than those in the diluted saliva samples (Figure 1B). The Cq values of the IPC in the serous saliva samples were significantly lower than those in the diluted saliva samples (Figure 1C).

### 3.2. Linear Regression Analysis of N1 and N2 Amplifications

**Figure 2:**
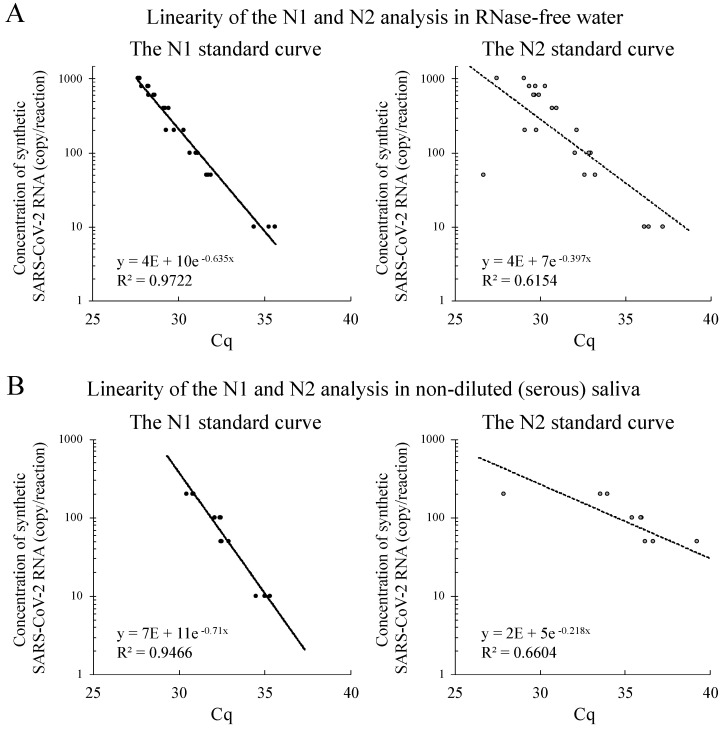
Linearity of the N1 and N2 amplifications in RNase-free water (RFW) and non-diluted saliva. Human saliva samples were collected from healthy volunteers. (**A**) N1 and N2 standard curves in RFW. (**B**) N1 and N2 standard curves in non-diluted saliva. Y axis indicates concentration of SARS-CoV-2 RNA STDs (1000, 800, 600, 400, 200, 100, and 50 to 10 cp/reaction; non-diluted saliva: 200, 100, and 50 to 10 cp/reaction), and X axis indicates the quantification cycles performed by LightCycler^®^ 96 software.

The linearity of the N1 and N2 Cq values in RFW and non-diluted saliva (clear serous saliva) is shown in Figure 2. The concentration gradients of the SARS-CoV-2 RNA STDs ranged from 1000 to 10 cp/reaction in RFW (Figure 2A) and from 200 to 10 cp/reaction in serous saliva (Figure 2B). The coefficients of determination (R^2^) of the N1 and N2 standard curves for the RFW were 0.972 and 0.615, respectively (Figure 2A). The R^2^ of the N1 and N2 standard curves for the serous saliva were 0.947 and 0.660, respectively (Figure 2B).

### 3.3. Detection Rates of N1 in Samples Containing Low Concentrations of SARS-CoV-2 RNA

**Figure 3:**
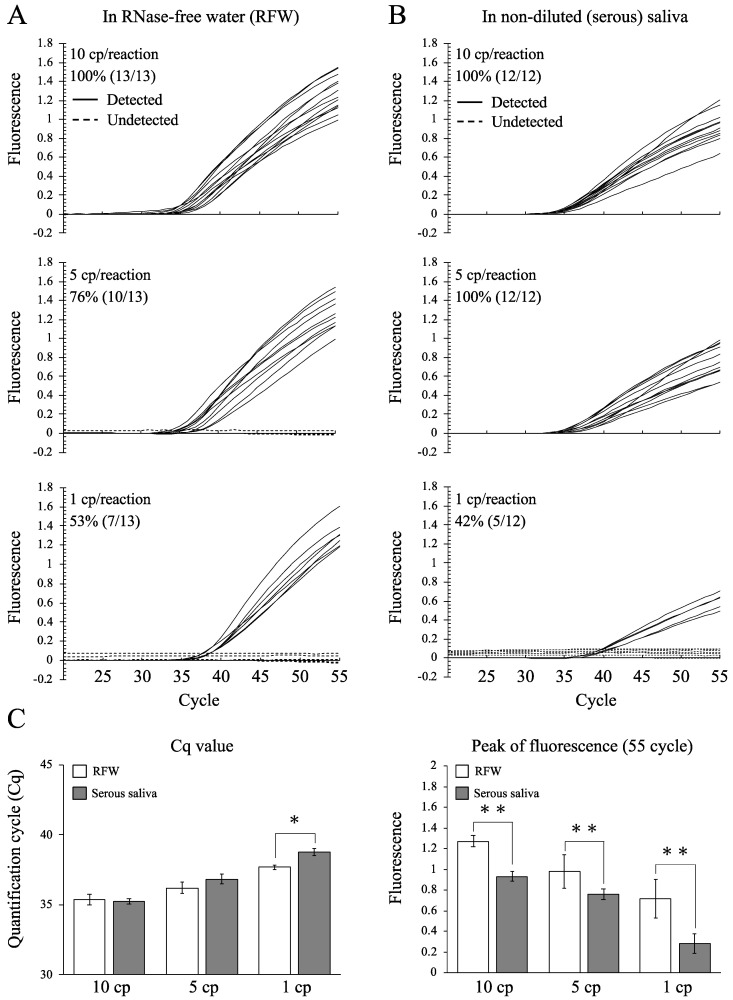
Detection rates of N1 in samples containing low concentrations of SARS-CoV-2 RNA STDs. (**A**) Detected (solid line) and undetected (dashed line) amplification curves of the N1 gene in RFW. (**B**) Detected (solid line) and undetected (dashed line) amplification curves of the N1 gene in non-diluted saliva. SARS-CoV-2 RNA STDs (10, 5, and 1 cp/reaction) were used for confirming the detection limit. Percentages indicate detection rates (detected samples/total samples). (**C**) Quantification cycle (Cq) and peak of fluorescence of the N1 gene in RFW (open column) and non-diluted saliva (grey column). Asterisks (* and **) indicate significant differences at *p* < 0.05 and 0.005 between the RFW and non-diluted saliva, respectively.

The amplification curves of the N1 gene representing low concentrations of the SARS-CoV-2 RNA STDs in RFW and non-diluted (clear serous) saliva are shown in Figure 3A,B, respectively. The detection rates of the N1 gene in RFW were 100% (13/13) at the 10 cp/reaction, 76% (10/13) at the 5 cp/reaction, and 53% (7/13) at the 1 cp/reaction levels (Figure 3A). The detection rates of the N1 gene in the serous saliva were 100% (12/12) at the 10 cp/reaction, 100% (12/12) at the 5 cp/reaction, and 42% (5/12) at the 1 cp/reaction levels (Figure 3B).

On comparing the Cq values and fluorescence intensities between RFW and saliva samples (Figure 3C), significant differences were observed. In terms of fluorescence intensities, significant differences were observed in 1, 5, and 10 cp/reaction levels. However, in Cq values group, a significant difference was observed only at the 1 cp/reaction level and no difference in the 5 cp/reaction and 10 cp/reaction levels between RFW and saliva samples. These results indicate that the Cq values of the N1 gene were comparatively stable, even when the sample conditions were variable. When using the calibration curve for predicting of virus amount (at the 1 cp/reaction level) in the RFW, it should be noticed that the predicted value of the virus amount will be lower than its true value (approximately 80% of the true value, Figure 3C).

Regarding the fluorescence intensities at all RNA STD concentrations (10, 5, and 1 cp/reaction), significant differences were observed between RFW and saliva samples. These results indicate that the fluorescence intensities were markedly reduced when a mixture with the RNA STDs and saliva samples was analyzed. This analytical condition was set as the ideal sample model. Fortunately, it was revealed that the Cq value may be more stable and useful than fluorescence intensity.

### 3.4. Detection Rates of N2 in Samples Containing Low Concentrations of SARS-CoV-2 RNA

**Figure 4:**
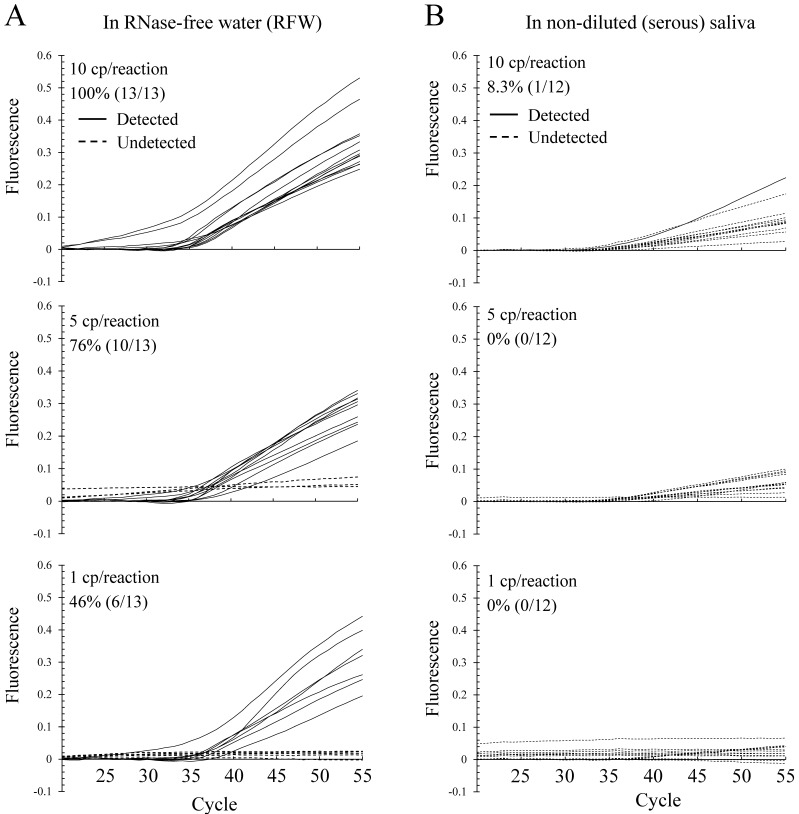
Detection rate of N2 in samples containing low concentrations of SARS-CoV-2 RNA. (**A**) Detected (solid line) and undetected (dashed line) amplification curves of the N2 gene in RFW. (**B**) Detected (solid line) and undetected (dashed line) amplification curves of the N2 gene in non-diluted saliva. SARS-CoV-2 RNA STDs (10, 5, and 1 cp/reaction) were used for confirming the detection limit. Percentages indicate detection rates (detected samples/total samples).

The amplification curves of the N2 gene representing low concentrations of the SARS-CoV-2 RNA STDs in RFW and non-diluted (clear serous) saliva are shown in Figure 4A,B, respectively. The detection rates of the N1 gene in RFW were 100% (13/13) at the 10 cp/reaction, 76% (10/13) at the 5 cp/reaction, and 46% (7/13) at the 1 cp/reaction levels (Figure 4A). The detection rates of the N2 gene in serous saliva were 8.3% (1/12) at the 10 cp/reaction, 0% (0/12) at the 5 cp/reaction, and 0% (0/12) at the 1 cp/reaction levels (Figure 4B).

## 4. Discussion

This study shows the stability of the Ampdirect™ 2019-nCoV detection kit for the quantitative analysis of SARS-CoV-2 RNA using human saliva samples. Our linearity evaluation clearly demonstrated a good quantitative ability for N1 gene analysis in both RFW and human saliva samples, even at low SARS-CoV-2 RNA concentrations (1000 to 10 cp/reaction) (Figure 2A,B). Additionally, high sensitivity detection of the N1 gene was indicated by PCR analysis of RFW and saliva samples containing low SARS-CoV-2 RNA concentrations (5 and 1 cp/reaction) (Figure 3A,B). Recently, the detection limits of SARS-CoV-2 RNA were reported using nucleotide extraction (approximately 10 cp/reaction) [4], direct qPCR (16-64 cp/reaction) [6] and LAMP (100 cp/reaction) [3] in saliva samples. In this study, similar and/or higher sensitivity was observed in saliva samples by direct PCR analysis using the Ampdirect™ 2019-nCoV detection kit in comparison with the other methods. To the best of our knowledge, detection at the level of 1 cp/reaction is one of the lowest for SARS-CoV-2 PCR analysis.

The marked difference observed between N1 and N2 may be explained by Rayleigh scattering, which refers to the scattering of light from small molecules in a liquid and can be extended to scattering from particles up to about a tenth of the wavelength of light. FAM (excitation: 495 nm, emission: 520 nm) can easily be influenced by particles of more than 0.05 µm in the samples. We assumed that even high-speed centrifugation at 20,000× *g* may not remove all small particles from the samples. Therefore, the shorter the wavelength, the easier it is for light to be scattered, because it tends to be scattered in inverse proportion to the fourth power of wavelength. The strong wavelength dependence of the scattering means that shorter wavelengths (i.e., FAM) are scattered more strongly than longer wavelengths (i.e., ROX) [20]. These results suggest that the fluorescence dye with a longer wavelength than FAM (excitation: 495 nm, emission: 520 nm) is more suitable for the direct PCR method.

The Cq value is a relatively stable index, even when comparing RFW and saliva samples (clear serous salivary fluids) (Figure 3C) under different conditions such as high and low viscosity. These results suggest one of the strongest advantages of the direct PCR analysis system. We assumed that standard curve construction with RFW could be useful in the calibration of viral concentrations in saliva samples and in estimating their viral numbers. Therefore, the present study suggests that direct PCR analysis is potentially useful in the quantitative analysis of SARS-CoV-2 even at very low concentration levels in human saliva.

This study further suggests that pretreatment of human saliva is one of the most important steps in real-time PCR analyses using a non-purification protocol because significant differences were observed in the fluorescence intensities and Cq values of the IPC when comparing diluted saliva versus serous saliva (Figure 1B,C). The fluorescence intensities of IPC amplifications in serous saliva were higher than those in diluted saliva, and the Cq values of IPC in serous saliva samples were significantly lower than those in diluted saliva, suggesting that the non-dilution method (i.e., using serous saliva) is more sensitive than the dilution method. We supposed that part of the salivary inhibiting factors for real-time PCR testing was removed by the high-speed centrifugation step in the non-dilution method. Therefore, our non-dilution method combined with high-speed centrifugation was suitable for quantitative real-time PCR analysis of low concentrations of salivary SARS-CoV-2.

## 5. Conclusions

This report demonstrated that the direct PCR analysis method using the Ampdirect™ 2019-nCoV detection kit enables the quantitative, real-time PCR analyses of SARS-CoV-2 concentrations in saliva samples without any RNA extraction and purification. This sensitive quantitative detection can contribute to restricting the spread of COVID-19 infection because it can detect low viral numbers, i.e., less than 10 cp/reaction, in saliva samples obtained from early-stage COVID-19 patients or asymptomatic individuals.

In a direct PCR method such as the Ampdirect™ 2019-nCoV detection kit, a clean final reaction mixture (containing a sample, sample denaturing solution, and PCR solution) is important to detect the viral RNA with high sensitivity and accuracy, especially when using saliva samples, because small particles such as small food residues easily scatter shorter wavelength light. Therefore, a fluorescence dye with a longer wavelength than FAM is recommended for the direct PCR method.

This study was conducted with the approval of the Ethics Committee of Shimadzu Techno-Research, Inc. Clinical experiments using patient samples could not be performed because this approval was limited to volunteers in the company. However, we analyzed a large number of serous saliva samples for clinical tests, and detected many positive samples using this method. Therefore, we hope that the pilot study described herein encourages any validation of our method by clinical researchers who may be interested in this simple and reliable method.

## Figures and Tables

**Table 1:** Reverse transcription and real-time PCR protocol.

Steps	Reactions	Cycles
Reverse transcription	42 °C for 600 s	1
Preincubation	95 °C for 60 s	1
2-Step amplification	95 °C for 5 s	45 or 55
	60 °C for 30 s	

N1 and N2 genes and the internal positive control (IPC) were measured by using the displayed PCR protocol.
